# Post-quantum cryptography for healthcare: securing medical data, connected devices, and digital health infrastructure

**DOI:** 10.3389/frhs.2026.1901282

**Published:** 2026-07-16

**Authors:** Sheng-Ping Wu, Sheng-Ding Wu, Kuo-Cheng Lu, Chia-Chao Wu

**Affiliations:** 1 Department of Electrical Engineering, National Taiwan Ocean University, Keelung, Taiwan; 2 Faculty of Medicine, School of Medicine, National Yang Ming Chiao Tung University, Taipei, Taiwan; 3 Division of Nephrology, Department of Medicine, Taipei Tzu Chi Hospital, Buddhist Tzu Chi Medical Foundation, New Taipei City, Taiwan; 4 Division of Nephrology, Department of Medicine, Tri-Service General Hospital, National Defense Medical University, Taipei, Taiwan

**Keywords:** crypto-agility, harvest-now-decrypt-later, healthcare cybersecurity, medical devices, ML-DSA, ML-KEM, post-quantum cryptography, quantum key distribution

## Abstract

Healthcare systems increasingly depend on interoperable electronic health records, picture archiving and communication systems, cloud analytics, remote monitoring, and connected medical devices. These workflows create a long-lived confidentiality problem: clinical, genomic, pediatric, psychiatric, and family-linked data may remain sensitive for decades, whereas the public-key algorithms that protect current transport and identity systems are vulnerable to future cryptographically relevant quantum computers. Shor's algorithm threatens RSA, finite-field Diffie-Hellman, elliptic-curve Diffie-Hellman, and elliptic-curve signatures, while Grover's algorithm reduces the effective security margin of symmetric primitives. This review validates the post-quantum transition pathway for healthcare and distinguishes software-deployable post-quantum cryptography from hardware-specialized quantum key distribution. We summarize the standardized NIST primitives, correct the status of Falcon/FN-DSA as a FIPS 206 algorithm still in development, analyze deployment constraints in Internet of Medical Things environments, and map cryptographic migration to healthcare governance. A staged, hybrid, crypto-agile migration strategy is recommended: prioritize long-confidentiality data, inventory cryptographic dependencies, deploy hybrid key establishment at enterprise gateways, reserve QKD for selected high-value fixed links, and require vendor update pathways for clinical devices.

## 1 Introduction

Healthcare delivery has moved from siloed local systems to a highly integrated, software-defined environment. Modern care depends on continuous exchange among electronic health records (EHRs), DICOM/PACS imaging repositories, laboratory information systems, genomic platforms, telemedicine services, cloud analytics, and artificial intelligence pipelines. HL7 FHIR formalizes RESTful data exchange for many clinical interfaces, while DICOM PS3.15 defines security and system-management profiles that reference transport-security mechanisms such as TLS for medical imaging workflows (1, 2).

This connectivity improves diagnostic access and continuity of care, but it also expands the attack surface across hospitals, cloud providers, device vendors, identity providers, and external research networks. Healthcare data breaches are frequent and clinically consequential, with hacking and IT incidents repeatedly identified as major breach vectors in the healthcare sector (3, 4).

Medical data require unusually long confidentiality lifetimes. Financial credentials can be reissued after a breach, but genomic information, pediatric histories, psychiatric records, reproductive histories, and family-linked phenotypes remain persistently identifying and scientifically informative. Genomic data are therefore treated as a high-risk data class in both privacy research and risk-management frameworks (5, 6). The post-quantum problem arises because a future cryptographically relevant quantum computer could run Shor's algorithm to solve integer factorization and discrete logarithm problems efficiently, undermining RSA, finite-field Diffie-Hellman, elliptic-curve Diffie-Hellman, and elliptic-curve digital signatures (7, 8). Grover's algorithm also provides a quadratic speed-up against brute-force search, which is why long-term systems should retain strong symmetric margins such as AES-256 rather than relying on minimum-strength symmetric keys (9).

Even if such a machine is not yet available, the healthcare threat is immediate because of harvest-now-decrypt-later (HNDL) risk: adversaries can capture encrypted traffic today and decrypt it later if the protected data remain valuable when quantum capabilities mature. CISA, NSA, and NIST therefore recommend quantum-readiness roadmaps, cryptographic inventories, risk assessment, and vendor engagement before a quantum-capable adversary exists (10, 11).

Two technologies are commonly discussed for quantum-era protection. Post-quantum cryptography (PQC) uses classical algorithms designed to resist both classical and quantum attacks and can be deployed through software and protocol updates. Quantum key distribution (QKD) uses quantum optical channels to distribute symmetric keys but requires specialized physical infrastructure and authenticated classical channels. In healthcare, PQC should be treated as the scalable enterprise baseline, while QKD is best reserved for selected high-assurance, fixed, point-to-point links where the operational and financial constraints are justified (12–19).

## 2 Methods

This article is a narrative, structured review intended to synthesize cryptographic, standards-based, and regulatory sources into actionable guidance for healthcare institutions; it is not a systematic review and does not follow PRISMA, as the objective is interpretive synthesis rather than quantitative pooling. Sources were identified through searches of PubMed, IEEE Xplore, ACM Digital Library, Scopus, and Google Scholar, supplemented by targeted retrieval of primary standards and authoritative guidance from NIST (FIPS 203-206, SP 800-208, SP 1800-38, IR 8547, and IR 8467), CISA and NSA, ETSI, the IETF (RFCs), the U.S. FDA, and applicable legal instruments (HIPAA and GDPR). Search terms combined cryptographic concepts (post-quantum cryptography, ML-KEM, ML-DSA, quantum key distribution, harvest-now-decrypt-later, and crypto-agility) with healthcare and device terms (electronic health record, DICOM/PACS, Internet of Medical Things, implantable device, and medical device cybersecurity). We prioritized finalized or in-progress standards from recognized bodies, peer-reviewed primary literature on algorithm security and performance, and authoritative regulatory and governance documents. Because the standardization landscape is evolving rapidly, the status of each algorithm was verified against primary NIST sources. Marketing materials and non-peer-reviewed vendor claims were excluded except where they were the only available source of deployment performance data, in which case they are identified as such.

## 3 Healthcare threat model

A clinically relevant post-quantum threat model should explicitly define the attacker, cryptographic target, data lifetime, and patient-safety implications.

**Attacker profile:** a state-sponsored actor, organized criminal group, or well-resourced insider-capable adversary able to conduct passive network interception across WAN links, cloud interconnects, partner networks, and vendor-managed device infrastructure.
**Near-term capability:** no quantum computer is available to the attacker, but large-scale storage and traffic collection are feasible. This phase creates HNDL exposure for long-lived data (10).
**Long-term capability:** the attacker eventually obtains or rents access to a fault-tolerant quantum computer capable of executing quantum algorithms relevant to public-key cryptanalysis. Published estimates vary by architecture and error-correction assumptions; the important clinical point is not a precise date or qubit count, but the mismatch between long data lifetimes and slow institutional migration (20).
**Primary confidentiality target:** encrypted clinical archives, genomic repositories, research data-sharing transfers, telemedicine sessions, and identity-provider traffic.
**Primary integrity target:** signatures used for software updates, medical-device firmware validation, certificate chains, audit records, and clinician identity assertions.
**The migration urgency can be summarized using Mosca's inequality:** if the time needed to discover vulnerable cryptography and migrate systems exceeds the time until a quantum-capable adversary becomes available, then data that still require confidentiality will be exposed. The mathematical vulnerability limits can be expressed dynamically through the relation:
  $$T_{discovery}+T_{migration}>T_{quantum}$$

**Figure 1:**
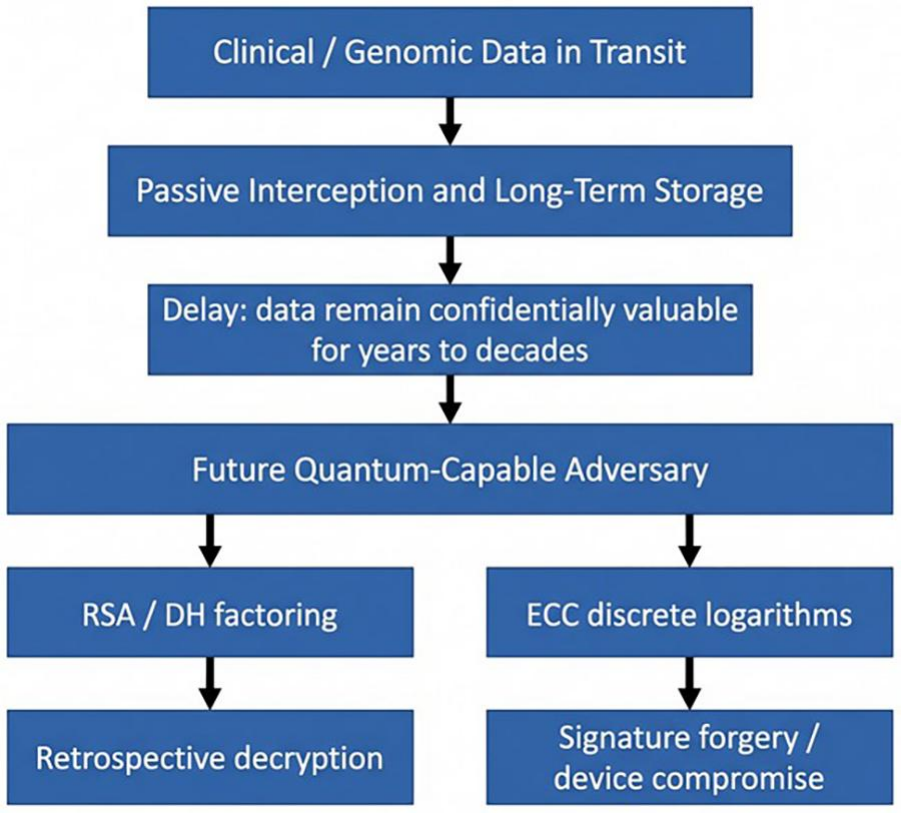
Harvest-now-decrypt-later risk pathway in healthcare networks. Schematic illustration of the temporal vulnerability in medical data security. The diagram outlines how long-lived clinical and genomic traffic intercepted today remains exposed to retroactive de Three-layer Quantum Key Distribution (QKD) deployment model for healthcare backbone links. Arccryption and signature forgery once a future cryptographically relevant quantum computer running Shor's or elliptic-curve discrete logarithm algorithms becomes operational.

Where ***T***_discovery_ is the time required to locate legacy primitives across the healthcare network, ***T***_migration_ is the operational timeline to deploy quantum-safe software, and ***T***_quantum_ is the arrival time of a CRQC. If the total migration timeline exceeds the quantum arrival target, all legacy-encrypted records with active confidentiality requirements are compromised (Figure 1).

Expert opinion on when a cryptographically relevant quantum computer (CRQC) will exist remains divided, and we do not attempt to adjudicate a date. Recent resource estimates have fallen sharply: a widely cited 2019 estimate of roughly 20 million noisy qubits over about eight hours for RSA-2048 was revised in 2025 to fewer than one million noisy qubits over about a week (21), with later analyses suggesting only low-thousands of logical qubits and vendor roadmaps projecting hundreds of logical qubits by the end of the decade. These figures are nonetheless theoretical or simulation-stage estimates rather than demonstrated factorizations; no CRQC currently exists, and maintaining fault-tolerant operation at the required scale and duration remains an unproven engineering problem that some researchers expect to persist for many years. For healthcare, the decision-relevant observation is robust to this uncertainty: because clinical and genomic confidentiality lifetimes routinely exceed a decade and institutional migration is slow, harvest-now-decrypt-later exposure is governed by the gap in Mosca's inequality rather than by any single forecast, so beginning cryptographic discovery and hybrid deployment now is justified under both optimistic and conservative timelines.

## 4 Cryptographic foundations of quantum-resistant primitives

### 4.1 Module-lattice-based key encapsulation: ML-KEM

NIST FIPS 203 standardizes ML-KEM, a module-lattice-based key-encapsulation mechanism derived from CRYSTALS-Kyber. Its security is related to the computational difficulty of Module Learning with Errors (MLWE), and NIST specifies three parameter sets: ML-KEM-512, ML-KEM-768, and ML-KEM-1024 (22).

For healthcare gateways, ML-KEM-768 is a practical default candidate because it aligns with NIST security category 3 and a 192-bit classical security target. ML-KEM-1024 may be appropriate for the most sensitive long-lived genomic or national-scale health repositories, but the additional key and ciphertext sizes should be evaluated against latency and interoperability constraints (22).

### 4.2 Module-lattice-based digital signatures: ML-DSA

NIST FIPS 204 standardizes ML-DSA, derived from CRYSTALS-Dilithium, for digital signatures. ML-DSA is the primary standardized post-quantum signature family for general-purpose authentication, software signing, and certificate-chain modernization (23).

For clinical identity services, the main constraint is not mathematical validity but operational overhead. For example, ML-DSA-65 has a 1,952-byte public key and a 3,309-byte signature, which is much larger than legacy elliptic-curve signatures. This inflation is manageable in high-bandwidth data transfers but can affect JSON Web Tokens, certificate chains, low-power networks, and constrained medical devices (23).

### 4.3 Stateless hash-based signatures: SLH-DSA

NIST FIPS 205 standardizes SLH-DSA, derived from SPHINCS+, as a stateless hash-based signature scheme. Because it relies on conservative hash-function assumptions rather than lattice assumptions, it provides algorithmic diversity, but it generally produces larger signatures and lower signing performance than ML-DSA. It is therefore best viewed as an important alternative or backup signature family rather than a universal replacement for all clinical authentication workflows (24).

### 4.4 Falcon/FN-DSA: corrected standardization status

Falcon is often discussed as FN-DSA because it produces compact signatures and is attractive for bandwidth-constrained settings. However, as of 23 May 2026, NIST describes FALCON as selected for publication in FIPS 206, with FIPS 206 still in development. It should therefore be cited as a selected or draft/pending NIST algorithm rather than as a finalized FIPS standard (25). Pre-standard Falcon documentation reports compact Falcon-512 public keys and signatures, but these values should not be presented as final FIPS 206 parameters until the standard is published (26).

### 4.5 Stateful hash-based signatures: XMSS and LMS

XMSS and LMS are stateful hash-based signature systems specified by RFC 8391 and RFC 8554 and approved for restricted use by NIST SP 800-208. These schemes can be appropriate for firmware signing, root trust anchors, and other low-frequency signing operations, but the signer must never reuse one-time signing state. State loss, backup duplication, or power-failure rollback can compromise the signature scheme (27–29).

Because security collapses if a one-time index is ever reused, stateful schemes must be engineered so that state can advance but never roll back. NIST SP 800-208 therefore restricts these schemes to environments in which key generation and signing occur inside a validated hardware cryptographic module that does not export the private key and that enforces non-reuse (29). In practice, institutions should implement four safeguards: first, write-ahead state advancement, in which the next one-time index and any associated tree state are durably committed to non-volatile, power-loss-safe storage before the signature is released, so that an interrupted operation can only skip indices and never repeat them; second, monotonic counters in hardware, such as a secure-element or HSM-backed counter that cannot be decremented, to anchor the index against tampering or filesystem manipulation; third, HSM-resident state management, keeping both the private key and its counter inside the module rather than in application memory or a database that could be snapshotted; and fourth, a strict prohibition on restoring signing state from backups, virtual-machine snapshots, or replicated copies, because any such restoration reuses already-consumed indices. These constraints make stateful signing a poor fit for mobile clinical carts and battery-powered endpoints subject to abrupt power loss; for such devices, on-device stateful signing should be avoided and signing centralized on a hardened HSM. Where multiple signing appliances must operate in parallel, the one-time index space should be partitioned across appliances using disjoint index ranges or distinct sub-trees, so that no two signers can ever draw the same index, rather than sharing a single counter (see Section 9, Phase 5). Where these guarantees cannot be assured operationally, the stateless scheme SLH-DSA (FIPS 205) should be preferred despite its larger signatures, since it removes the state-management failure mode entirely.

## 5 QKD as a complementary, not universal, healthcare control

QKD protocols such as BB84 and entanglement-based schemes are grounded in quantum measurement disturbance and the no-cloning theorem (12–14). Modern QKD security analyses address practical device imperfections, side channels, finite-key effects, and long-distance or networked deployment constraints, while measurement-device-independent variants reduce certain detector-side vulnerabilities (15–18, 30–32).

In a healthcare architecture, QKD does not encrypt EHR or DICOM payloads directly. Instead, it distributes symmetric keys to conventional encryptors, which then protect application traffic with standard symmetric encryption. ETSI GS QKD 014 defines a REST-based key-delivery API between secure application entities and key-management entities, supporting integration between QKD key-generation infrastructure and conventional network-security equipment (19).

**Figure 2:**
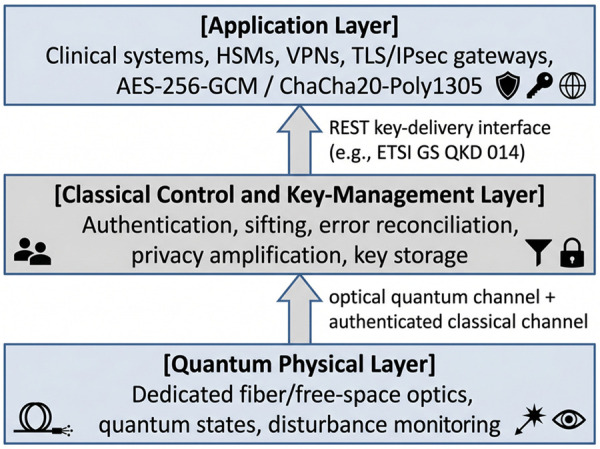
Three-layer Quantum Key Distribution (QKD) deployment model for healthcare backbone links. Architectural overview of high-assurance link protection. The diagram displays the integration of the physical quantum channel (bottom layer) and the classical control/key-management layer (middle layer) with conventional enterprise network applications and symmetric cryptographic primitives via the REST-based ETSI GS QKD 014 key-delivery interface (top layer).

QKD has important deployment limits: it requires dedicated or carefully engineered optical links, trusted nodes or advanced network designs for extended distances, specialized hardware, and robust operational monitoring. Therefore, QKD is best positioned as a high-assurance layer for fixed, high-value links such as a hospital datacenter connected to a genomic repository, not as a replacement for enterprise-wide PQC deployment (Figure 2).

## 6 Algorithmic parameters and resource constraints

Table 1 summarizes practical parameters relevant to healthcare deployment. The table distinguishes finalized NIST FIPS standards from selected or stateful schemes, correcting the common citation error of treating FN-DSA/Falcon as already finalized (Table 1).

**Table 1:** Algorithmic parameters, standardization Status, and resource constraints of quantum-resistant primitives in healthcare environments.

Primitive	Status/source	Class	Security category	Public key/encapsulation key	Secret key/signature/ciphertext overhead	Healthcare implication
ML-KEM-768	Final: NIST FIPS 203 (22)	KEM; module-lattice MLWE	Level 3	1,184 B	Decapsulation key 2,400 B; ciphertext 1,088 B	Practical default for hybrid enterprise key exchange.
ML-KEM-1024	Final: NIST FIPS 203 (22)	KEM; module-lattice MLWE	Level 5	1,568 B	Decapsulation key 3,168 B; ciphertext 1,568 B	Candidate for highest-sensitivity long-lived data paths.
ML-DSA-65	Final: NIST FIPS 204 (23)	Signature; module-lattice	Level 3	1,952 B	Secret key 4,032 B; signature 3,309 B	Good general-purpose signature option, but JWTs and certificates expand.
SLH-DSA	Final: NIST FIPS 205 (24)	Stateless hash-based signature	Multiple parameter sets	Varies by parameter set	Larger signatures than ML-DSA in many configurations	Useful for algorithmic diversity; evaluate size/performance carefully.
FN-DSA/Falcon	Selected; FIPS 206 in development (25)	Signature; NTRU lattice/Falcon family	Expected Level 1 and Level 5 sets	Falcon-512 public key about 897 B in pre-standard documentation (26)	Falcon-512 signature about 666 B in pre-standard documentation (26)	Promising for bandwidth-constrained settings, but cite as pending rather than final.
XMSS/LMS	RFC 8391, RFC 8554, NIST SP 800–208 (27–29)	Stateful hash-based signature	Parameter-dependent	Compact public keys	Signatures commonly measured in kilobytes	Suitable for controlled firmware/root signing when state management is reliable.

Comprehensive evaluation of standardized and selected post-quantum cryptography (PQC) algorithms alongside stateful hash-based signature schemes. This table distinguishes finalized NIST Federal Information Processing Standards (FIPS 203, 204, and 205) from draft/pending protocols (FIPS 206/FN-DSA) and details respective public key, secret key, signature, and ciphertext overheads to evaluate their practical deployment implications for clinical enterprise workflows and low-power medical devices.

### 6.1 Internet of medical things bottlenecks

Connected medical devices such as implantable monitors, continuous glucose monitors, infusion systems, bedside sensors, and home telemetry hubs often operate under strict constraints on RAM, flash, radio bandwidth, firmware updateability, and battery life. PQC migration must therefore be engineered differently for enterprise servers and for Internet of Medical Things (IoMT) endpoints.

The main bottleneck is rarely a single cryptographic operation in isolation. Instead, the risk comes from packet fragmentation, repeated retransmission, larger certificates, larger signatures, and longer radio-on time. IEEE 802.15.4 networks have very small link-layer frames compared with conventional Ethernet, and 6LoWPAN must use compression and fragmentation to carry IPv6 packets over such networks (33, 34).

For example, an ML-DSA-65 signature of 3,309 bytes cannot fit into one constrained wireless frame. In protocols such as 6LoWPAN, fragmentation increases the probability that a single packet loss forces retransmission of a much larger logical message. This affects battery drain, latency, and reliability. For safety-critical devices, a conservative design should minimize on-device PQC operations, use gateway-assisted handshakes where appropriate, and reserve large signatures for firmware validation or infrequent trust-establishment events rather than continuous telemetry.

Empirical benchmarks support the view that the binding constraint in constrained medical devices is communication and timing rather than raw computation. On the ARM Cortex-M4, which NIST recommended as a microcontroller optimization target and which underlies the pqm4 benchmarking framework (35), ML-KEM outperforms classical ECDH and ML-DSA remains within an order of magnitude of ECDSA (36), so the dominant cost is the larger key and signature objects rather than the arithmetic itself. On lower-end Cortex-M0 + devices, ML-KEM keys and ciphertexts fit easily within typical static RAM, but ML-DSA signing exhibits large tail latency, with reported 99th-percentile signing times of roughly 0.5 s for ML-DSA-44 and over 1 s for ML-DSA-87, which is problematic for hard-real-time telemetry (37). Healthcare-specific evaluations report modest but non-trivial overheads, on the order of an 11% increase in computation in one integrated Internet of Medical Things framework (38), together with comparisons of NewHope, Kyber, and XMSS on wearable hardware across encryption and decryption time, memory, and power consumption (39). Taken together, these results justify reserving large post-quantum signatures for infrequent firmware-validation and trust-establishment events, using gateway-mediated handshakes for routine traffic, and prioritizing ML-KEM-based key establishment over on-device signing in battery-limited endpoints.

### 6.2 Hybrid key establishment during migration

**Figure 3:**
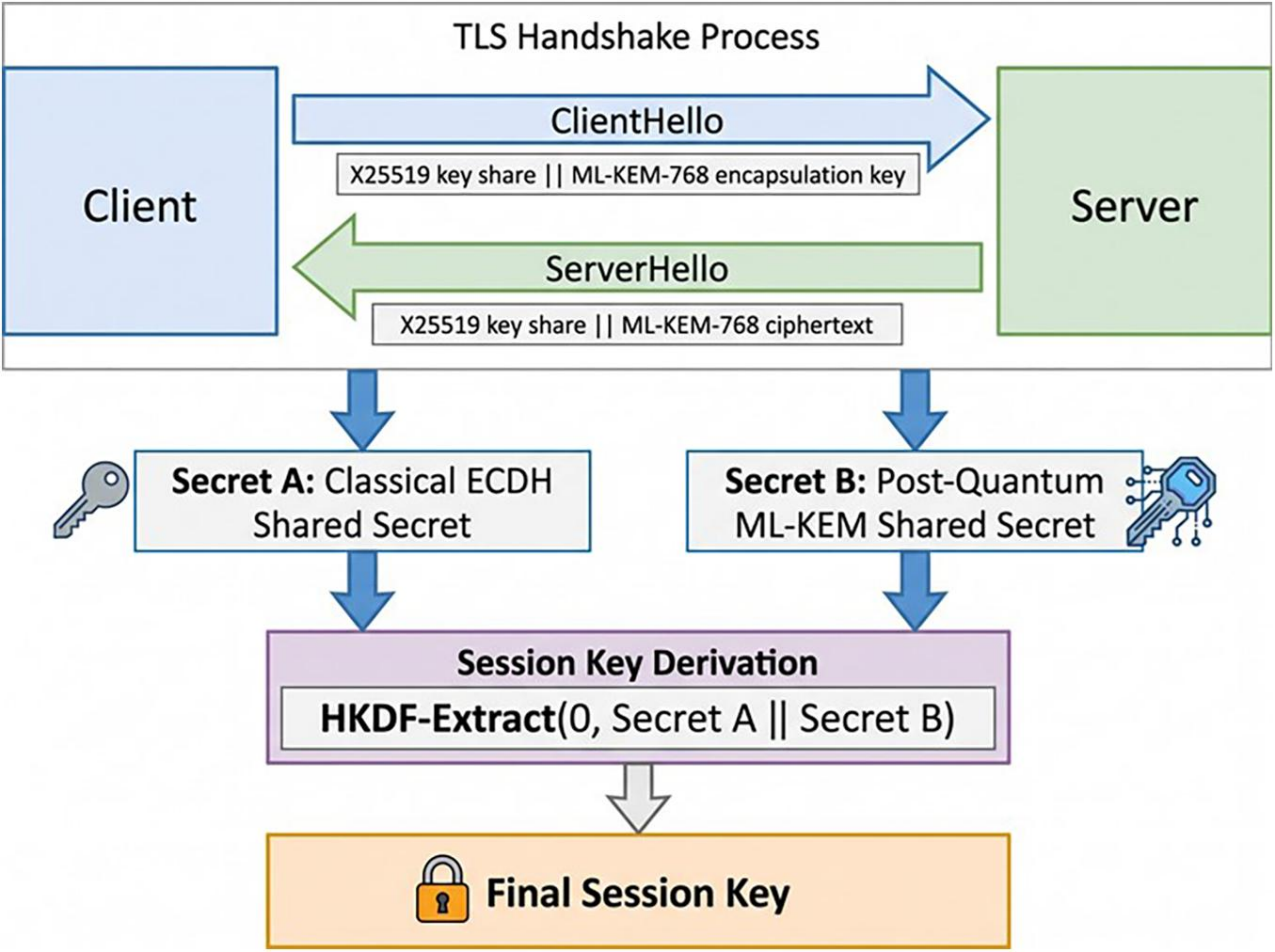
Hybrid TLS handshake with classical ECDH and post-quantum ML-KEM Key exchange. Data flow diagram of a transitional crypto-agile handshake process between a client and server. The figure demonstrates the concurrent transmission of a classical X25519 key share and a post-quantum ML-KEM-768 encapsulation key/ciphertext, which are subsequently combined via a key-derivation function [$HKDF\text(-)Extract$] to produce a secure session key.

During transition, hybrid key establishment can combine a classical scheme such as X25519 with ML-KEM-768 in a single handshake. The resulting session key is derived from both shared secrets using a key-derivation function. This approach maintains protection if one component remains secure and gives institutions time to migrate certificates, hardware security modules, middleware, and regulated devices (8, 11, 22) (Figure 3).

Hybridization should not be treated as a substitute for governance. It must be accompanied by cryptographic discovery, downgrade-resistance testing, certificate-chain planning, library validation, and monitoring to ensure systems do not silently fall back to classical-only modes.

The performance cost of hybrid key establishment is modest but should be planned for explicitly, because it is concentrated in connection establishment rather than in steady-state data transfer. The dominant cost is communication rather than computation: an X25519 plus ML-KEM-768 ClientHello carries a combined key share of about 1,216 bytes, comprising a 1,184-byte ML-KEM-768 encapsulation key and a 32-byte X25519 share, compared with roughly 32 bytes for classical X25519 alone, so the larger handshake messages may span additional TCP segments (40). Reported protocol-level latency increases are small, on the order of 1 ms of additional median handshake time in controlled TLS 1.3 measurements and frequently within measurement noise (41), and production data indicate only a low-single-digit reduction in throughput in the worst case in which every request performs a fresh full handshake, for example an approximately 2.3% reduction in transactions per second for hybrid X25519 with ML-KEM-768 when connection reuse is disabled (42). The operational implication for high-frequency HL7 FHIR RESTful endpoints is therefore clear: because the penalty is incurred per handshake, gateways should amortize it through TLS session resumption, persistent connections and connection pooling, and HTTP/2 multiplexing, so that the post-quantum cost is paid once per session rather than once per request and per-request overhead approaches the classical baseline. Terminating and reusing hybrid TLS at the enterprise gateway, rather than renegotiating on every FHIR call, keeps the additional cost within clinically acceptable bounds while preserving harvest-now-decrypt-later protection on the wire.

## 7 Clinical data lifecycle risk stratification

PQC migration should be prioritized by confidentiality lifetime, legal exposure, and patient-safety consequence. Not all clinical data carry the same quantum risk. Short-lived telemetry that loses value within hours has lower HNDL exposure than genomic repositories, pediatric longitudinal records, and cross-generational research cohorts (Table 2).

**Table 2:** Clinical data lifecycle risk stratification and recommended cryptographic controls.

Lifecycle phase	Examples	Quantum-era risk level	Recommended control priority	Key citation
Data generation	Bedside vitals, transient sensor data, device telemetry	Low to medium; depends on sensitivity and retention	Use strong transport security; avoid overloading constrained devices with unnecessary large signatures.	(3, 33, 34)
Real-time transmission	FHIR APIs, telemedicine, DICOM transfer, remote monitoring	Medium to high for long-lived records in transit	Deploy hybrid PQC key establishment at gateways and cloud ingress points.	(1, 2)
Primary storage	EHR archives, PACS archives, clinical identity records	High	Prioritize cryptographic inventory, storage encryption, key-management modernization, and PQC-ready certificates.	(8, 10, 11)
Research sharing	Genomic data, biobanks, AI model-training datasets	Very high	Prioritize PQC-protected transfer and long-term key-management controls.	(5, 6)
Archival/destruction	Long-term pediatric records, family-linked genomic histories	Critical when retention exceeds cryptographic transition timeline	Apply quantum-safe protection before external sharing or long-term archive movement.	(6, 10, 11)

Matrix mapping individual phases of the clinical data lifecycle—ranging from real-time generation and transmission to permanent archival or destruction—against quantum-era confidentiality risk levels. The table details specific recommended technical controls, prioritizing high-consequence, long-confidentiality assets such as biobanks and genomic repositories over transient telemetry.

## 8 Governance, compliance, and healthcare regulation

Current healthcare laws generally do not name PQC as a mandatory algorithmic requirement. They impose risk-based duties to protect confidentiality, integrity, availability, and patient safety. Therefore, PQC migration is best framed as a foreseeable risk-management response rather than as a direct statutory command (Table 3).

**Table 3:** Major legal, regulatory, and cryptographic governance frameworks relevant to post-quantum healthcare migration.

Framework	Relevant provision or guidance	Validated interpretation	PQC-relevant governance implication	Citation
HIPAA Security Rule	45 CFR 164.312 technical safeguards; encryption/decryption and transmission security are addressable implementation specifications (46).	HIPAA requires risk-based technical safeguards for ePHI; it does not currently prescribe PQC algorithms.	Covered entities should document quantum risk in security risk analysis where long-lived ePHI is affected.	(46)
EU GDPR	Article 9 special categories; Article 32 security of processing, including encryption and risk-appropriate measures (47).	Health, genetic, and biometric data are special-category data; Article 32 supports risk-based encryption and resilience.	PQC readiness can be justified for high-risk long-retention datasets, especially genomic and research archives.	(47)
FDA medical-device cybersecurity guidance and FD&C Act Section 524B	Premarket submissions for cyber devices must include cybersecurity information, including vulnerability management and updates.	FDA emphasizes secure design, updates, and lifecycle risk management; it does not currently require a formal CBOM named as such.	Manufacturers should provide crypto-agility evidence, SBOM/cryptographic dependency information, and update pathways for PQC migration.	(43)
NIST/CISA/NSA quantum-readiness guidance	Roadmaps, cryptographic inventories, risk assessment, and vendor engagement.	Quantum readiness is a migration program, not a single algorithm replacement.	Healthcare institutions should create cryptographic inventories and prioritize long-confidentiality assets.	(8, 10, 11)
NIST genomic data profile	NIST IR 8467 s public draft; structured cybersecurity and privacy risk management for genomic data.	Genomic data require tailored cybersecurity and privacy controls across processing and sharing.	Genomic pipelines should be Tier 1 assets for PQC migration.	(6)

Regulatory analysis evaluating international health data and device security frameworks, including the US HIPAA Security Rule, EU GDPR, US FDA medical-device cybersecurity guidelines, and joint NIST/CISA/NSA guidance. The table provides validated clinical interpretations and risk-management implications for maintaining compliance during institutional post-quantum migration programming.

## 9 Implementation roadmap for healthcare institutions

A realistic post-quantum migration program should minimize patient-safety disruption while reducing HNDL exposure. The following staged roadmap is recommended.

Several institutional barriers shape any realistic migration program. Legacy systems are pervasive in healthcare: imaging modalities, laboratory analyzers, and embedded controllers frequently run on operating systems and cryptographic libraries that are no longer maintained and that cannot accept post-quantum certificates or larger key and signature objects without firmware replacement. Budget constraints compound this, because cryptographic migration competes with clinical capital priorities and rarely generates direct revenue; cost is driven less by algorithms, which are royalty-free, than by inventory discovery, testing, certificate-authority modernization, hardware-security-module replacement, and device re-validation. Interoperability across vendors is a recurring obstacle, because a hybrid handshake or post-quantum certificate is usable only if every endpoint in a clinical transaction, including the EHR, the gateway, the imaging device, the identity provider, and third-party integrations, negotiates compatible parameters; divergent vendor timelines can force institutions to maintain classical and hybrid stacks in parallel for extended periods. Workforce limitations are significant, as few healthcare security teams include staff trained in applied cryptography, and cryptographic inventory and downgrade-resistance testing require skills that must often be acquired or contracted. Finally, regulatory considerations specific to medical devices constrain the pace of change, because software changes to regulated devices may trigger re-validation or premarket review obligations, so post-quantum updates must be planned within each manufacturer's quality-management and update framework rather than applied *ad hoc* (43). These barriers motivate the staged, hybrid, gateway-first sequence described below, which concentrates early effort where institutions have the most control and defers on-device changes until vendor update pathways and validation evidence are available.

Because the standardized algorithms are royalty-free, the marginal cost of migration is dominated by labor and re-validation rather than licensing; institutions can reduce total cost by sequencing migration so that discovery and gateway-level hybrid deployment, where reuse is highest, precede device-by-device replacement.

### 9.1 Phase 1: cryptographic discovery and inventory

Create a cryptographic inventory across applications, certificates, VPNs, EHR interfaces, PACS/DICOM services, FHIR gateways, cloud workloads, HSMs, mobile applications, device firmware, and third-party vendor connections. This inventory should include algorithm, key length, certificate authority, expiration date, library version, hardware dependency, and owner. NIST SP 1800-38B provides a practical starting point for cryptographic discovery (11).

### 9.2 Phase 2: data-lifetime and clinical-risk classification

Classify assets by confidentiality lifetime and patient-safety impact. Genomic repositories, pediatric longitudinal records, identity infrastructure, firmware-signing keys, and research-sharing pipelines should be treated as high-priority assets because they combine long sensitivity with large-scale consequences if compromised.

### 9.3 Phase 3: hybrid enterprise baseline

Deploy hybrid key establishment at perimeter gateways, VPNs, API gateways, and high-value internal service boundaries before attempting full replacement across all devices. Hybrid deployment allows interoperability testing and reduces the risk of sudden clinical workflow disruption (8, 11, 22).

### 9.4 Phase 4: signature modernization and certificate planning

Plan for ML-DSA or alternative standardized signature schemes in certificates, token signing, firmware-signing pipelines, and code-signing systems. Evaluate token and certificate size inflation before deploying post-quantum signatures into latency-sensitive clinical identity systems.

### 9.5 Phase 5: constrained-device engineering

For IoMT environments, avoid assuming that enterprise PQC profiles can be copied directly into devices. Use gateway mediation, staged firmware updates, minimized signature frequency, and controlled stateful signatures only where operational state can be protected. Safety validation must include packet-loss, power-failure, and rollback testing.

### 9.6 Phase 6: targeted QKD feasibility

Assess QKD only for fixed, high-value links where dedicated fiber, link distance, trusted-node architecture, operational staffing, and key-management integration are feasible. QKD should complement, not replace, PQC across the wider clinical enterprise (19).

### 9.7 Phase 7: continuous crypto-agility governance

**Figure 4:**
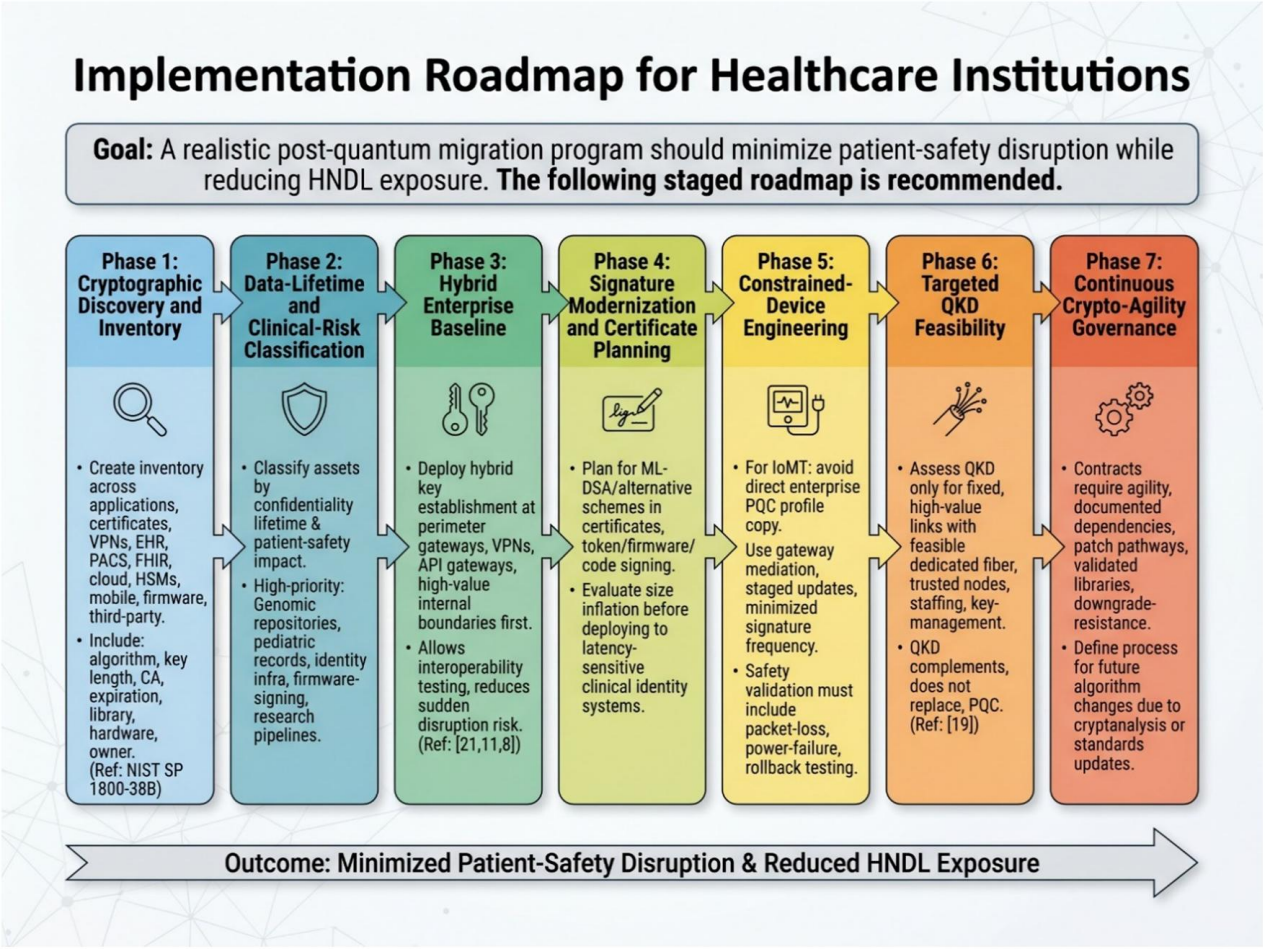
Implementation roadmap for healthcare institutions to support post-quantum migration. The staged framework outlines seven phases: cryptographic discovery and inventory, data-lifetime and clinical-risk classification, hybrid enterprise baseline, signature modernization and certificate planning, constrained-device engineering, targeted quantum key distribution feasibility, and continuous crypto-agility governance. The overall goal is to minimize patient-safety disruption while reducing hardware and network dependency exposure (HNDL exposure).

Procurement and vendor contracts should require crypto-agility, documented cryptographic dependencies, patch pathways, validated libraries, downgrade-resistance testing, and end-of-life plans for quantum-vulnerable algorithms. Governance should also define how post-quantum algorithms are changed if future cryptanalysis or standards updates require migration (Figure 4).

To make crypto-agility enforceable in regulated purchasing, procurement teams and vendor contracts should require evidence against the following criteria, which can be expressed and audited in a machine-readable Cryptographic Bill of Materials (CBOM) using the OWASP CycloneDX standard and its associated open tooling (44, 45):

1. Cryptographic Bill of Materials (CBOM). Require a machine-readable CBOM, for example in OWASP CycloneDX v1.6 format, enumerating every algorithm, key type and parameter, protocol, certificate, and implementing library in the product, with dependency mapping to components, and updated and re-delivered with each release.
2. Quantum-vulnerability disclosure. Require explicit identification within the CBOM of all quantum-vulnerable public-key usage, including RSA, finite-field and elliptic-curve Diffie-Hellman, and ECDSA, together with the migration status of each.
3. Crypto-agility evidence. Require demonstration that algorithms can be replaced without hardware replacement, including configurable cipher suites, parameterized key sizes, and the ability to install new certificates and signature algorithms by update.
4. Software-defined patching lifecycle. Require a documented, authenticated, and reversible update mechanism with signed firmware, a stated patch cadence and end-of-support date, and demonstrated capacity to deliver cryptographic updates over the supported lifetime of the device.
5. Post-quantum migration roadmap. Require a written plan and timeline for adopting NIST-standardized algorithms (ML-KEM, ML-DSA, and where appropriate SLH-DSA), including support for hybrid key establishment during transition.
6. Downgrade resistance and validation. Require evidence of downgrade-resistance testing and the use of validated cryptographic libraries, with contractual notification obligations if the product silently falls back to classical-only modes.
7. Regulatory alignment. For medical devices, require that cryptographic update pathways and the CBOM or SBOM be consistent with the manufacturer's premarket cybersecurity documentation and quality-management obligations (43).

Embedding these as contract clauses and acceptance-test criteria allows healthcare institutions to make crypto-agility a condition of purchase rather than a post-deployment retrofit.

## 10 Limitations

This review has limitations. As a narrative rather than a systematic review, source selection involved expert judgment and may not capture every relevant study, although we mitigated this by anchoring on authoritative standards and primary literature. A formal economic analysis was beyond our scope; we note qualitatively that the principal costs of migration arise from cryptographic discovery, testing, certificate and hardware-security-module modernization, and device re-validation rather than from algorithm licensing, and that quantifying these costs in real clinical settings is an important direction for future work. Finally, the field currently offers few peer-reviewed, end-to-end case studies of post-quantum deployment in production healthcare environments; the implementation evidence cited here derives largely from device-level benchmarks and prototype architectures, and prospective institutional case studies are needed to validate the proposed roadmap at scale.

## 11 Conclusion

Post-quantum migration is an immediate governance priority for digital healthcare because the confidentiality lifetime of medical and genomic data can exceed the expected stability of today's public-key cryptography. The most scalable path is a tiered architecture in which PQC becomes the software baseline for enterprise systems, while QKD is reserved for selected high-assurance optical links. Healthcare organizations should begin with cryptographic discovery, risk classification by data lifetime, hybrid key establishment at enterprise gateways, and vendor requirements for crypto-agility.
